# Determinants of Male Involvement in the Prevention of Mother‐to‐Child Transmission of HIV in the Bamenda Health District, Cameroon

**DOI:** 10.1155/jotm/9721872

**Published:** 2026-07-27

**Authors:** Victorine Mulur Ngachangong, Charles Falang Doumta, Dickson Shey Nsagha, Emmanuel Yenshu Vubo

**Affiliations:** ^1^ Department of Nursing and Midwifery, The University of Bamenda, Bamenda, Northwest Region, Cameroon, unibda.net; ^2^ Department of Public Health and Hygiene, University of Buea, Buea, Southwest Region, Cameroon, ubuea.cm; ^3^ Department of Sociology and Anthropology, University of Buea, Buea, Southwest Region, Cameroon, ubuea.cm

**Keywords:** determinants, HIV/AIDS, MTCT, PMTCT

## Abstract

**Background:**

Prevention of mother‐to‐child transmission (PMTCT) of HIV remains a major public health problem. Male involvement has been seen as one of the ways of scaling up the PMTCT of HIV. However, studies have shown a low male involvement; hence, the objective of this study was to assess the determinants of male involvement in the PMTCT of HIV.

**Method:**

The study was a community‐based cross‐sectional study in the Northwest Region of Cameroon. A total of 406 participants from the Bamenda Health District were selected using a consecutive sampling method. Data were collected through a well‐structured questionnaire. Data entry and analysis were performed using SPSS Version 23. The results were presented at a 95% confidence interval.

**Results:**

The level of good knowledge, attitudes, and practices of PMTCT of HIV by men was 86.5%, 75.4%, and 42.6%, respectively. The knowledge of mother‐to‐child transmission was high at 98.5% (CI: 96.8–99.3) and significantly associated with education and MI (*p*∼0.001). Men who had attained tertiary and secondary education were more likely to have a higher MI (OR: 0.37, 95% CI: 0.2–0.7; 0.49, 95% CI: 0.2–0.49). Those who were married (OR: 0.17, 95% CI: 0.05–0.6) or living together (OR: 0.39, 95% CI: 0.2–0.7) were less likely to have a high male involvement in PMTCT.

**Conclusion:**

Although the men were highly knowledgeable and had a positive attitude toward PMTCT, this was not translated into practice, as less than 50% of the men were involved in PMTCT.

## 1. Introduction

HIV/AIDS continues to be a disease of Public Health importance and is one of the world’s most serious and development challenges. About 88 to 94.1 million people have been infected since the start of the HIV/AIDS epidemic [1, 2]. The recent global HIV statistics show that 40.8 million people are currently living with HIV, with approximately 1.3 million people becoming newly infected with HIV [2–4]. Africa was the hardest hit, with about 50% of the new infections [3, 5]. In 2024, among the people living with HIV (PLWH), 1.4 million were children within 0–14 years, and about 53% of PLWH were women and girls [2–5]. The 2024 statistics showed that 45% of the infections were among women and girls, which poses the problem of mother‐to‐child transmission (MTCT). Although there has been a decrease in new infections in children, progress has been slow, as only 63% knew their status, 87% of whom had access to antiretroviral (ARV) therapy, and 86% had achieved viral suppression [3, 5].

Cameroon has seen a steady decrease in HIV prevalence from 5.4% (2004) to 2.7% (2018) and further to 2.3% in 2024, driven by improved testing and prevention programs [6–8]. However, PMTCT coverage remains suboptimal, with only 34.2% of children under 10 receiving ART [8]. The transmission rate among exposed infants at 6–8 weeks also dropped from 3.22% to 1.78% in 2024. However, only 34.2% of children under 10 years old are receiving ARV treatment, and 32.4% of adolescents aged 10–19 years. The youths between 15 and 49 years did not even understand HIV transmission, while only 55% of women aged 15–24 were tested for HIV [8].

Key preventive measures involve preventing HIV infection among women of reproductive age, preventing unwanted pregnancies among HIV‐infected women by enhancing the use of contraceptives, prevention of transmission from HIV‐infected women to their infants by voluntary counseling and testing (VCT) for HIV during pregnancy, and for positive women, provision of ARV drug prophylaxis during delivery, and to their neonates, with support to their families [9]. HIV pretest and posttest counseling, the use of ARTs, and support services through pregnancy, delivery, and breastfeeding are key preventive measures among HIV‐positive women [10, 11]. According to Chi and colleagues [12], ARV use has greatly reduced transmission from mother to child, especially in breastfeeding mothers.

Success in the reduction of MTCT of HIV depends not only on the use of ARTs but also on continuing support from male partners. Studies have shown that male partner involvement in PMTCT activities reduces the risk of transmission and even death compared to those not involved [10, 13, 14]. It facilitates ART initiation and adherence and ensures hospital birth by mothers and also safer feeding choices [10, 15, 16], thereby reducing dropout from PMTCT programs, thus reducing MTCT of HIV, enhancing uptake of HIV testing, thereby impacting the survival of infants born to HIV‐positive mothers [14, 16–18].

Despite global progress, male participation in PMTCT remains low in sub‐Saharan Africa, influenced by cultural, economic, and health system barriers [16, 19–21]. Key strategies for improvement include enhancing knowledge and attitudes. Unfortunately, there are few studies on men’s knowledge of, attitudes toward, and involvement in PMTCT in Cameroon. Therefore, this study aimed to assess the determinants, such as knowledge, attitudes, and practices, of men regarding the PMTCT of HIV in certain communities within the Bamenda Health District (BHD), to develop strategies for increasing male partner involvement in antenatal care (ANC) and PMTCT.

## 2. Method

### 2.1. Study Design/Study Setting

The research employed a cross‐sectional community‐based descriptive study design. It was carried out in the North West Region, specifically the BHD, which has 17 health areas. The Azire Health Area, Nkwen Rural, and Nkwen Baptist Health Areas were purposively selected for this study because they are easily accessible and make up 44.7% of the total population of the BHD.

### 2.2. Study Population/Sampling

The study population included men aged 20 years and older who had at least one child and provided consent. The sample size was calculated using a single population proportion formula (Cochran’s formula) with the following assumptions: a 95% confidence interval (CI), a 5% margin of error, and an estimated proportion of 50% (0.5), since the proportion of MI in PMTCT in BHD was unknown. This resulted in a sample size of 384, and with a 10% nonresponse rate, the final sample size was 422.

A consecutive and convenient sampling method was used to recruit the participants into the study. Convenience sampling was used due to accessibility constraints, which may limit generalizability to more remote or less health‐engaged populations. The participants were recruited from communities in these health areas that were easy to access, some of the hospitals found within these areas, and those who accompanied their partners for ANC or IWC, churches, and social meeting groups.

### 2.3. Data Collection

#### 2.3.1. Definition and Measurement of Male Involvement

Challenges have arisen in assessing male partner involvement in PMTCT because there is no standard definition of what it entails. One major challenge is developing a way to measure male involvement that captures both the practical support men provide to women and how they challenge societal gender norms [22]. Various indicators have been proposed to assess male partner involvement in PMTCT, such as communication between spouses (disclosure of HIV status), ANC attendance, childbirth, antenatal testing, support during ANC/pregnancy, and couple ARV treatment [22, 23]. As a result, some studies have combined these indicators into a composite score to broadly define male involvement in PMTCT.

Male involvement in PMTCT lacks a standardized definition. In this study, MI was operationalized as a composite measure including: (1) initiating HIV discussions during pregnancy, (2) requesting partner HIV testing, (3) accompanying partner to ANC visits, (4) providing financial support, (5) participating in couple counseling/testing, and (6) supporting safe infant feeding decisions. This aligns with prior frameworks by Malindi and Maputle [15] and Amano et al. [24]. The study by Triulzi et al. [25] defined male partner involvement in PMTCT as attending ANC, participating in couple HIV testing, meeting final obligations, and providing social and psychological support. This illustrates that the definition varies across different studies, but key elements generally include participating in ANC, couple HIV testing, and fulfilling financial obligations. The qualitative study by Triulzi et al. [26] identified two areas of male involvement or support for women in PMTCT, which were accompanying women to and providing access to transport to the clinic. The complexity of defining male partner involvement in PMTCT could be seen in Mukuni et al. [27], where male partner involvement was based on the 10‐item male partner involvement scale developed by Hampanda et al. in 2020. It included: (1) attendance at health care visits during pregnancy or postpartum; (2) encouraging facility delivery; (3) reminders to take HIV medication; (4) reminders to go for HIV or PMTCT care; (5) giving transport money to go to the clinic/dispensary; (6) reminders to give infant prophylaxis; (7) help giving infant prophylaxis; (8) collecting HIV medications for the women or infant; (9) encouraging specific infant feeding behavior; (10) encouraging infant testing.

Data were collected using a questionnaire adapted from those of the studies [22, 24]. A well‐structured questionnaire was used to collect data for each of the specific objectives. First, questions on knowledge of HIV transmission, MTCT of HIV, and PMTCT of HIV, questions on attitudes toward PMTCT, and lastly, practices of PMTCT. Knowledge was assessed using 20 questions divided into 3 sections (HIV transmission, MTCT, PMTCT), attitude was assessed using a 5‐point Likert scale containing 12 questions, and male partner involvement was assessed using 12 questions.

The data were collected by trained assistants, and coordination and supervision of the data collection process were ensured. For knowledge, attitude, and practice, internal consistency of items was checked using Cronbach’s alpha (α), and a score of 0.729 was obtained.

A knowledge, attitude, and practice questionnaire was administered to men within the BHD to assess their knowledge, attitudes toward, and practices of male involvement in PMTCT. The questionnaire consisted of 44 questions or statements, 20 questions on HIV transmission, MTCT, and reduction of MTCT; 12 questions on attitudes; and 12 questions on male partner involvement. The knowledge questions offered 3 possible answers (yes, no, and I don’t know). Each correct answer scored 1 point, while each incorrect answer received a score of 0. For knowledge, the total score was 20, and a score of 13 or higher indicated adequate knowledge of PMTCT, while a score below 13 indicated inadequate knowledge.

The attitude component was scored using a 5‐point Likert scale (1 = *strongly agree*, 2 = *agree*, 3 = *neutral*, 4 = *disagree*, and 5 = *strongly disagree*). The highest possible score was 5, and the lowest was 1. The attitude questions had a total score of 60, and a score of 40 or higher was considered positive, while a score below 40 indicated a negative attitude.

Meanwhile, for male involvement, we had 3 possible responses to the questions (yes, no, and I don’t know). A correct response was scored 1 point, while a wrong answer, or “I don’t know,” was scored zero. For male involvement in PMTCT, a score of 8–12 was considered to indicate that the participant was involved in the PMTCT of HIV, and a score less than 8 indicated the person was not involved.

Bloom’s cutoff point has been widely adopted to assess KAP studies in health in categorizing knowledge, attitude, and practice. The cutoffs are as follows: 80%–100% (good KAP), 60%–79% (moderate KAP), and less than 60% (poor KAP), as seen in other studies [28]. Knowledge, attitude, and practice scores were categorized using Bloom’s cutoff criteria, modified to 66.6% for this community‐based sample to avoid overexclusion. This threshold aligns with similar KAP studies in LMICs where higher cutoffs (≥ 80%) may not reflect real‐world understanding [28, 29].”

#### 2.3.2. Data Analysis

The data were coded and entered into a computer software Statistical Package for the Social Sciences (SPSS) Version 21, where cleaning and data analysis were done. Descriptive statistics were used to determine levels of knowledge, attitudes, and male involvement in PMTCT. Results were presented at 95% CI, and *p* values less than 0.05 were considered statistically significant.

#### 2.3.3. Data Quality Assurance

The data collectors were trained by the principal investigator to ensure data quality. The questionnaires were pretested, and questions were reviewed for clarity, understandability, flow, and structure, with modifications made as needed. The principal investigator oversaw the entire data collection process.

### 2.4. Ethical Considerations

Ethical approval for the study was obtained from the Faculty of Health Sciences at the University of Buea, the Institutional Review Board (2019/1023B‐07/UB/SG/IRB/FHS), the Regional Hospital Bamenda Review Board (069/APP/RDPH/RHB/IRB), and the Cameroon Baptist Convention Health Services Review Board (IRB2020‐09). Administrative authorizations were secured from the Regional Delegation of Public Health in Bamenda—Northwest Region, the Regional Hospital Bamenda, Nkwen Baptist Hospital, and the Azire Integrated Health Center. This was followed by signed consent forms from participants after they received information about the study’s objectives and expected outcomes at each stage of data collection. Neither the patients nor the public were involved in the design, conduct, reporting, or dissemination of the study results.

## 3. Results

### 3.1. Sociodemographic Characteristics of Study Participants

Out of the 422 male participants recruited for the study, 406 responded, with a response rate of 96%. The age varied between 21 years and 63 years, with a mean age of 35.5 years and a ± SD of 8.8 years.

The majority were within the age range of 21–41 years (78.8%), of whom 72.7% were married. Most of the participants were living together (80.2%), and close to half (47.1%) had obtained a tertiary level of education. Results showed that 80.3% of the participants were employed, and 65.3% had a standard source of income. Participants were principally businessmen (36.9%) and civil servants (33.7%). The mode for participants with children ranged from 1 to 2 years (41.6%) and 3–5 years (38.4%). Slightly more than half of the participants lived in rural areas (53.7%) (Table 1).

**TABLE 1:** Demographic characteristics of male participants in some Health Areas in the Bamenda Health District from June to September 2020.

Variables	Parameters	Frequency *n* (%)
Age range (years)	21–30	151 (37.2)
	31–41	169 (41.6)
	≥ 42	86 (21.2)
	Total	406 (100)

Marital status	Married	295 (72.7)
	Cohabiting	89 (21.9)
	Unmarried	16 (3.9)
	Divorced	6 (1.5)
	Total	406 (100)

Living together	Yes	328 (80.8)
	No	78 (19.2)
	Total	406 (100)

Level of education	Primary	59 (14.5)
	Secondary	156 (38.4)
	Tertiary	191 (47.1)
	Total	406 (100)

Employment status	Employed	326 (80.3)
	Unemployed	80 (19.7)
	Total	406 (100)

Standard source of income	Yes	265 (65.3)
	No	141 (34.7)
	Total	406 (100)

Occupation	Farmer	61 (15.0)
	Business	150 (36.9)
	Civil servant	137 (33.7)
	Others	58 (14.3)
	Total	406 (100)

Number of children	1–2	168 (41.6)
	3–5	155 (38.4)
	≥ 6	81 (20.0)
	Total	406 (100)

The age range of the last child	≤ 1	142 (36.0)
	2–5	181 (44.1)
	6–10	45 (11.0)
	> 10	36 (8.9)
	Total	406 (100)

Residence	Urban	188 (46.3)
	Rural	218 (53.7)
	Total	406 (100)

### 3.2. Men’s Knowledge of PMTCT

Results showed that the participants were highly knowledgeable, with 86.5% having adequate knowledge of PMTCT of HIV (Supporting Figure 1).

### 3.3. Knowledge of HIV Transmission and MTCT of HIV

Results showed that the majority of the participants knew the modes of HIV transmission: having sexual intercourse with an infected person, sharing contaminated sharps/needles, and blood transfusion (92.1%, 91.9%, and 90.6%). Almost all of the participants (98.5%) said HIV could be transmitted from mother to child, especially during breastfeeding, labor and delivery, and pregnancy (81.2%, 74.3%, and 60.5%, respectively) (Supporting Table 1).

### 3.4. Knowledge of the Reduction of MTCT of HIV

A total of 92.3% of participants cited HIV counseling and testing for pregnant women as a way of reducing MTCT of HIV, while 76.1% mentioned counseling of male partners. Four‐fifths of the participants (80.8%) mentioned the early initiation of ARV therapy, and more than three‐fifths (61.6%) said complete avoidance of breastfeeding. 72.6% had heard of the PMTCT program, and 62.6% said it was offered in all government/private hospitals. Most (91.7%) knew pregnant women were being counseled and tested at ANC. The overall knowledge of PMTCT was high (86.5%) (Supporting Table 1).

#### 3.4.1. Association Between Sociodemographic Characteristics and Knowledge

The association showed that educational level and religion influence knowledge on PMTCT, with a statistically significant *p* of less than 0.05 (*p*∼0.001; 0.004, respectively). It was also seen to be associated with MI (*p*∼0.001). (Table 2).

**TABLE 2:** Association between sociodemographic characteristics, attitude, male involvement, and knowledge.

Variable	Knowledge		*p* value
	Correct	Incorrect	
Age range (years)			
20–30	130 (86.1%)	21 (13.9%)	0.401
31–41	143 (84.6%)	26 (15.4%)	
≥ 42	78 (90.7%)	8 (9.3%)	
Marital status			
Married	261 (88.5%)	34 (11.5%)	0.049
Cohabiting	70 (78.7%)	19 (21.3%)	
Unmarried	20 (90.9%)	2 (9.1%)	
Number of children			
One	67 (84.8%)	12 (15.2%)	0.888
Two	108 (87.1%)	16 (12.9%)	
> Two	176 (86.7%)	27 (13.3%)	
Educational level			
Primary	40 (67.8%)	19 (32.2%)	0.001
Secondary	132 (85.2%)	23 (14.8%)	
Tertiary	179 (93.2%)	13 (6.8%)	
Occupation			
Farmer	49 (80.3%)	12 (19.7%)	1.27
Business	126 (84%)	24 (16%)	
Civil servant	122 (89.1)	15 (10.9%)	
Others	54 (93.1%)	4 (6.9%)	
Religion			
Christian	297 (88.7%)	38 (11.3%)	0.004
Muslim	23 (85.2%)	4 (14.8%)	
Others	31 (70.5%)	13 (29.5%)	
Attitude			
Positive	264 (75.2%)	87 (24.8%)	0.502
Negative	42 (76.4%)	13 (23.6%)	
Male involvement			
Involved	165 (95.4%)	8 (4.6%)	∼0.001
Not involved	186 (79.8%)	47 (20.2%)	

#### 3.4.2. Attitudes to PMTCT

**FIGURE 1:**
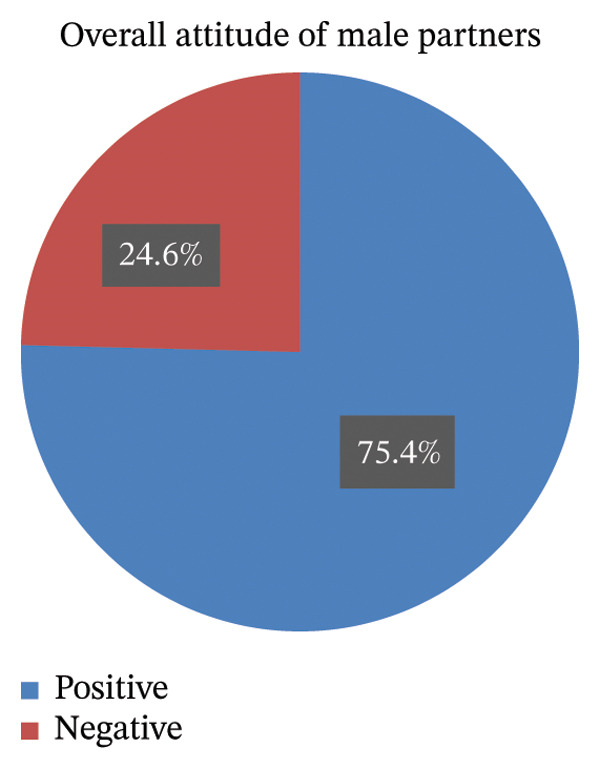
Overall attitude of male partners on PMTCT of HIV at the Bamenda Health District from June to September 2020.

Of the 406 participants, 75.4% had a positive attitude toward male involvement in the PMTCT, while 24.6% had a negative attitude (Figure 1).

The participants portrayed positive attitudes in various areas, such as agreeing to accompany their partners to ANC/PMTCT (82.5%); pregnant women should be tested for HIV even if their partner disagrees (76.6%), 80% agreed the fact that couples should be tested together for HIV during ANC follow‐up. On the other hand, they were not too sure/disagreed that the use of condoms reduces the chances of MTCT of HIV (46.3%), while 39% agreed to or were either neutral to postponing HIV testing to postdelivery, as pregnancy is stressful (Supporting Table 2).

#### 3.4.3. Practices of PMTCT Among Men

Participants identified the following key areas in MI in the PMTCT of HIV: asking their partners about information from ANC (72.4%), reminding their spouses of their ANC schedule (78.1%), and 85.4% usually covering all the medical expenses of ANC, while 61.8% said they would request the HIV test for their pregnant partners. Of the 64.6% of the participants who had at least accompanied their partners once for ANC, 53.4% entered the ANC room with their partners. Out of the 47.5% who were counseled during their spouse’s pregnancy, 80.6% were counseled and tested together; 92.9% revealed their results to each other. More than three‐fifths (63.3%) said they did not use condoms with partners during pregnancy. The overall level of MI in PMTCT was 42.6% (Supporting Table 3).

#### 3.4.4. Binary Logistic Regression Between Sociodemographic Characteristics and Male Involvement in PMTCT

Results showed a significant association between male involvement in PMTCT and marital status (OR: 0.17, 95% CI: 0.05–0.6), and living together (OR: 0.39, 95% CI: 0.2–0.7), who were less likely to be involved in the PMTC. Men with a high educational level (OR: 0.37, 95% CI: 0.2–0.7, OR: 0.46, 95% CI: 0.2–0.9) and who had a standard source of income (OR: 0.58, 95% CI: 0.4–0.9) were less likely to use PMTCT services. Men with correct knowledge were 5.2 times more likely to be involved in PMTCT (OR: 5.2, 95% CI: 2.4–11.4). (Table 3).

**TABLE 3:** Binary logistic regression between sociodemographic characteristics and male involvement in PMTCT.

Variable	OR	95% CI	*p* value
Age range (years)			
20–30	1.69	(0.98–2.9)	0.058
31–41	1.04	(0.6–1.8)	0.888
≥ 42	ref		
Marital status			
Married	0.17	(0.05–0.6)	0.005
Cohabiting	0.36	(0.1–1.3)	0.162
Unmarried	Ref		
Living together			
Yes	0.39	(0.2–0.7)	0.001
No	Ref		
Number of children			
One	0.857	(0.4–1.8)	0.680
Two	1.03	(0.5–2.0)	0.916
> Two	ref		
Educational level			
Primary	Ref		
Secondary	0.46	(0.2–0.9)	0.021
Tertiary	0.37	(0.2–0.7)	0.003
Standard source of income			
Yes	0.58	(0.4–0.9)	0.011
No	ref		
Employment Status			
Unemployed	0.76	(0.5–1.2)	0.194
Employed	Ref		
Occupation			
Farmer	1.4	(0.6–2.8)	0.436
Business	0.91	(0.5–1.7)	0.758
Civil servant	0.69	(0.4–1.3)	0.237
Others	ref		
Religion			
Christian	1.1	(0.7–2.0)	0.831
Muslim	0.6	(0.2–1.6)	0.312
Others	ref		
Knowledge			
Correct	5.21	(2.4–11.4)	∼ 0.001
Incorrect	Ref		

## 4. Discussions

### 4.1. Knowledge of PMTCT Among Men

This study was aimed at understanding the knowledge, attitudes, and practices of male partners’ involvement in the PMTCT of HIV. The results of this study showed a high level of knowledge (80.2%) on HIV transmission, MTCT, and ways of reducing HIV transmission among pregnant women. It was higher than the 39.3% and 46.7% in Nigeria and Ethiopia, respectively [22, 30]. The study aligns with that of Alemayehu and Co [29] in Ethiopia, where more than three‐quarters of the men demonstrated adequate knowledge of PMTCT. Reasons for the increase in knowledge could include increased ANC attendance, greater education on the importance of male involvement, the attachment of other services to ANC, and the use of invitation letters.

The men knew the routes of MTCT of HIV, and this confirmed the study of Alemayehu and Co [29] where men were able to identify the routes of MTCT: during pregnancy, labor and delivery, and breastfeeding (78%, 95.5%, and 95.5%, respectively) but contrary to Harrison and Co [30] in Nigeria where only 43.2% correctly identified transmission during pregnancy and 30.2% during labor. This may be because of exposure to health talks on the PMTCT of HIV; the study in Nigeria was carried out among military men.

Most of the men said PMTCT services were available in government hospitals, with the major activity being VCT, confirming the studies of others [29, 30]. Results showed that those who had a tertiary level of education were more likely to have higher knowledge of PMTCT, which was similar to studies carried out in Ethiopia and Uganda [29, 31, 32]. The same conclusion could not be made for religion.

### 4.2. Men’s Attitude to the PMTCT of HIV

The men showed a positive attitude (75.4%) toward male involvement in PMTCT. This was demonstrated in the following areas: pregnant women should be accompanied by their male partners for ANC and should be tested for HIV even if the male partner disagreed with the testing (unlike in some cultures where the woman is expected to get permission from the husband before such is done). This was in line with other studies where the participants had an overall positive attitude (83.8%), and wives did not need their husbands’ approval to attend ANC/PMTCT or get HIV tested [22, 29]. Accompanying pregnant women to ANC/PMTCT clinics and getting tested with them was supported, and such clinics were not meant for women and children only, as was seen in other studies [22, 33]. The positive attitude may be due to greater sensitization of men to ANC/PMTCT services.

A positive attitude also implies that an HIV‐positive woman and her unborn baby will receive adequate support, thus preventing divorce or separation, as fear of losing one’s partner has been identified as a barrier to male involvement in PMTCT. Finally, a positive attitude can be attributed to the fact that more women are being educated and the changing societal roles.

### 4.3. Male Partner Involvement in the PMTCT of HIV

The level of male partner involvement in PMTCT was assessed using a series of questions, and the result put the level of MI in PMTCT at 42.6%, which was high compared to that of Byamugisha et al. [23] in Uganda, where MI was 26% and 21% in a study carried out in Buea, South West Region, Cameroon [17]. Comparatively, MI in PMTCT in this study was also high compared to that of other studies, as presented by Kelembo and Co [20] in Malawi. MI in PMTCT was 12.5% in Northern Tanzania, 16% in Kenya, and 24% in the Ivory Coast, respectively. It was also higher than the result obtained by Amano and colleagues in North West Ethiopia, where MI in PMTCT was 20.9% (24). Comparatively, MI was lower than the results obtained by Orne–Gliemann and Co., as seen in Kelembo [20]. The relatively high MI in PMTCT could be attributed to the fact that there is increased sensitization of men and more men attending ANC, where health talks are given on such issues, as could be seen from the study, that 64.8% of the men said they had accompanied their female partners at least once for ANC. Moreover, most of the men were within the age range of 20–40 years, which can make it more likely for them to attend ANC.

Most of the men identified the following key areas in MI in the PMTCT of HIV: The majority said they will ask their partner about information from ANC, three‐quarters said they always remind their spouses of their ANC schedule, while most (85.2%) said they usually cover all the medical expenses of ANC. Knowledge of PMTCT enhances male partner involvement in PMTCT, as men who ask their partners for information from ANC follow‐up are more likely to participate in its activities [24], which was similar to our findings.

The study showed that most men (64.8%) had at least once accompanied their partners to ANC, with 53.2% entering the ANC room with their partners. In contrast, Haile and Brhan’s study [29] found that only 21% of men accompanied their wives to ANC, but 82.1% tested together for HIV. In Cameroon, traditional gender roles often designate ANC as a “woman’s domain,” which may explain why married and cohabiting men showed lower involvement. Qualitative studies in similar settings reveal that men may perceive ANC attendance as undermining their masculinity or as unnecessary if the woman is healthy [33, 34]. While 47.5% of the men were counseled during their wife’s pregnancy, Adane and collaborators’ study [31] reported only 10.1% of men had been tested; however, 80.6% were counseled and tested together, and 93% shared their results. This suggests that when couples undergo counseling and testing together, acceptance of results tends to increase, promoting the acceptance of ARV therapy and encouraging medical care for the unborn, thus helping to reduce pediatric HIV. The study by Alemayehu and Co [29] indicated that men were aware that women were counseled and tested at the ANC/PMTCT clinic.

Reasons for low male involvement in PMTCT may be due to the busy nature of men, stigma, and culture, etc., which have been seen as barriers to PMTCT. Qualitative studies in similar settings reveal that men may perceive ANC attendance as undermining their masculinity or as unnecessary if the woman is healthy [32–34]. When men are involved in voluntary testing and counseling, adherence will be enhanced. The study of Tigabu and Dessie showed that three‐quarters (76.3%) of the women said their husbands/partners were tested for HIV/AIDS during their ANC follow‐up and that 204 (91.1%) shared their HIV test results with their partners/husbands [35].

More than half of the participants said they did not use condoms with partners during pregnancy for preventive purposes. The low condom use during pregnancy (36.7%) contrasts with high knowledge of MTCT prevention. This may reflect low perceived risk, trust in seronegative status, or cultural resistance to condom use within marriage. Interventions should address relational dynamics and promote dual protection strategies. Similarly, in the study by Byamugisha [23], 61% of the women had not asked their partners if they needed to use condoms to prevent transmission of HIV. Contrarily, half (52.6%) of the men in a study in the Enebsiesarmider District, North West, Ethiopia, were willing to use condoms during sexual intercourse for PMTCT of HIV [31]. This could be due to awareness of the preventive measures of MTCT of HIV and also because the study was carried out among HIV‐positive couples. Women who are counseled and tested together with partners with a positive test result are more likely to use condoms for protection [15].

Finally, there was a significant association between knowledge and MI in PMTCT, with a P ∼ 0.001, indicating that having the correct knowledge was linked to a higher likelihood of male partner involvement. Men who were married and living together were less likely to be involved in PMTCT, possibly because they view ANC as a woman’s domain. Men who had attained secondary and tertiary education and had a standard source of income were more likely to get involved in PMTCT. These results were similar to those of Harrison et al. [30] and Byamugisha et al. [23], where educated men had a higher male involvement index than those with primary education. Studies have shown that the more people are educated, the more likely they are to have good practices and positive health‐seeking behavior. A standard source of income also provides easy access to health care and supports positive health‐seeking behavior, which will enhance the PMTCT of HIV.

## 5. Conclusion

Based on the findings, it can be concluded that the men’s knowledge of PMTCT was high, with 86.5% having correct knowledge.

Most of them had a positive attitude toward male partner involvement in PMTCT.

Though knowledge and attitudes were high, this was not translated into practice, as less than half of the men were involved in PMTCT. We therefore recommend that (1) mandatory couple booking systems and ANC invitation letters for male partners should be implemented at the health system level, (2) conduct male‐focused PMTCT education through community leaders, peer‐educators in workplaces, and at the community level, and (3) at the policy level, gender sensitive PMTCT training should be integrated into the national HIV guideline.

### 5.1. Limitations

This study used a cross‐sectional design and convenience sampling, which limits causal inference and generalizability. Self‐reported data may be subject to social desirability bias. Future research should employ longitudinal or mixed‐methods approaches to explore the sociocultural determinants of MI in depth.

**Nomenclature**
ANC: Antenatal care
IRB: Institutional Review Board
LMIC: Low‐middle‐income countries
MTCT: Mother‐to‐child transmission
PMTCT: Prevention of mother‐to‐child transmission of HIV.

## Author Contributions

All authors made substantial contributions to the conception, design, acquisition of data, analysis, and interpretation. They contributed to the drafting of the article and critically revised it for important intellectual content.

## Funding

The study was partly funded by the Denise and Lenora Foundation.

## Conflicts of Interest

The authors declare no conflicts of interest.

## Supporting Information

Additional supporting information can be found online in the Supporting Information section.

## Supporting information


**Supporting Information** Figure 1: Knowledge Level of male partners on PMTCT of HIV at the Bamenda Health District from June to September 2020. Supporting Table 1: Knowledge of PMTCT. Supporting Table 2: Attitudes of Men on PMTCT. Supporting Table 3: Practices.

## Data Availability

All relevant data on the research will be made available on request.
