# Supplementary material for: Determinants of Male Involvement in the Prevention of Mother‐to‐Child Transmission of HIV in the Bamenda Health District, Cameroon
Source: J Trop Med. 2026 Jul 27;2026:9721872. doi: 10.1155/jotm/9721872 (PMC13402937; doi:10.1155/jotm/9721872)
Supplement: Supplementary file 1 — Supporting Information Figure 1: Knowledge Level of male partners on PMTCT of HIV at the Bamenda Health District from June to September 2020. Supporting Table 1: Knowledge of PMTCT. Supporting Table 2: Attitudes of Men on PMTCT. Supporting Table 3: Practices. [file JOTM-2026-9721872-s001.zip › Supplemental figure 1.docx]

**SUPPLEMENTAL FIGURE ONE**

**Level of Knowledge on PMTCT**

**Figure 1: Knowledge Level of male partners on PMTCT of HIV at the Bamenda Health District from June to September 2020**
