# Supplementary material for: Determinants of Male Involvement in the Prevention of Mother‐to‐Child Transmission of HIV in the Bamenda Health District, Cameroon
Source: J Trop Med. 2026 Jul 27;2026:9721872. doi: 10.1155/jotm/9721872 (PMC13402937; doi:10.1155/jotm/9721872)
Supplement: Supplementary file 1 — Supporting Information Figure 1: Knowledge Level of male partners on PMTCT of HIV at the Bamenda Health District from June to September 2020. Supporting Table 1: Knowledge of PMTCT. Supporting Table 2: Attitudes of Men on PMTCT. Supporting Table 3: Practices. [file JOTM-2026-9721872-s001.zip › Supplemental Table 1 - Knowledge.docx]

**SUPPLEMENTAL TABLE 1 – KNOWLEDGE OF PMTCT**

**Knowledge of Male participants on PMTCT in the Bamenda Health District from June to September 2020**

| **Variable** | **Parameter** | **N (%)** | **95% CI** | |
| --- | --- | --- | --- | --- |
|  |  |  | **Lower** | **Upper** |
| **HIV Transmission** |  |  |  |  |
| Unprotected sexual intercourse with an infected person | Correct | 374 (92.1) | 89.1 | 94.4 |
|  | Incorrect | 32 (7.9) | 5.6 | 10.9 |
|  | Total | 406 (100) |  |  |
| Eating from the same plate | Correct | 325 (80.0) | 75.9 | 83.7 |
|  | Incorrect | 81 (19.6) | 16.3 | 24.1 |
|  | Total | 406 (100) |  |  |
| Sharing contaminated sharps/needles | Correct | 373 (91.9) | 88.8 | 94.1 |
|  | Incorrect | 33 (8.1) | 5.8 | 11.2 |
|  | Total | 406 (100) |  |  |
| Blood transfusion with infected blood | Correct | 368 (90.6) | 87.4 | 93.1 |
|  | Incorrect | 38 (9.4) | 6.9 | 12.6 |
|  | Total | 406 (100) |  |  |
| **Mother-to-Child Transmission** |  |  |  |  |
| HIV can be transmitted from mother to child | Correct | 400 (98.5) | 96.8 | 99.3 |
|  | Incorrect | 6 (1.5) | 0.6 | 3.2 |
|  | Total | 406 (100) |  |  |
| Transmission during pregnancy | Correct | 242 (60.5) | 55.6 | 65.2 |
|  | Incorrect | 158 (39.5) | 34.8 | 44.4 |
|  | Total | 400 (100) |  |  |
| Transmission during labour and delivery | Correct | 297 (74.3) | 69.8 | 78.3 |
|  | Incorrect | 103 (25.7) | 21.7 | 30.3 |
|  | Total | 400 (100) |  |  |
| Sleeping with the baby on the same bed | Correct | 322 (80.5) | 76.3 | 84.1 |
|  | Incorrect | 78 (19.6 | 15.9 | 23.7 |
|  | Total | 400 (100) |  |  |
| During Breastfeeding | Correct | 325 (81.2) | 77.1 | 84.8 |
|  | Incorrect | 75 (18.8) | 15.2 | 22.9 |
|  | Total | 400 (100) |  |  |

| **Reduction of MTCT of HIV** |  |  |  |  |
| --- | --- | --- | --- | --- |
| HIV counseling and testing for pregnant mothers | Correct | 375 (92.3) | 89.4 | 94.6 |
|  | Incorrect | 31 (7.7) | 5.4 | 10.6 |
|  | Total | 406 (100) |  |  |
| HIV counseling and testing of male partners | Correct | 309 (76.1) | 71.7 | 80.0 |
|  | Incorrect | 97 (23.9) | 20.0 | 28.7 |
|  | Total | 406 (100) |  |  |
| Antiretroviral drugs for the infected mother and child | Correct | 332 (81.8) | 77.7 | 85.2 |
|  | Incorrect | 74 (18.2) | 14.8 | 22.3 |
|  | Total | 406 (100) |  |  |
| Delivery by Cesarean section | Correct | 199 (49.0) | 44.2 | 53.9 |
|  | Incorrect | 207 (51) | 46.1 | 55.8 |
|  | Total | 406 (100) |  |  |
| Complete avoidance of breastfeeding | Correct | 250 (61.6) | 56.8 | 66.2 |
|  | Incorrect | 156 (38.4) | 33.8 | 43.2 |
|  | Total | 406 (100) |  |  |
| Exclusive breastfeeding | Correct | 236 (58.1) | 53.3 | 62.8 |
|  | Incorrect | 170 (41.9) | 37.2 | 46.7 |
|  | Total | 406 (100) |  |  |
| Use of contraception | Correct | 101 (24.9) | 20.9 | 29.3 |
|  | Incorrect | 305 (75.1) | 70.7 | 79.1 |
|  | Total | 406 (100) |  |  |
| Heard of the PMTCT Program | Correct | 326 (80.3) | 76.2 | 83.4 |
|  | Incorrect | 80 (19.7) | 16.1 | 23.9 |
|  | Total | 406 (100) |  |  |
| Offered in all government/private health services | Correct | 244 (74.8) | 69.9 | 79.3 |
|  | Incorrect | 82 (25.2) | 20.8 | 30.1 |
|  | Total | 326 (100) |  |  |
| Pregnant women are counseled and tested at ANC | Correct | 374 (92.1) | 89.1 | 94.4 |
|  | Incorrect | 32 (7.8) | 5.6 | 10.9 |
|  | Total | 406 (100) |  |  |
| Early initiation of Antiretroviral therapy for child | Correct | 331 (81.5) | 77.5 | 85.0 |
|  | Incorrect | 75 (18.5) | 11.0 | 22.5 |
|  | Total | 406 (100) |  |  |
| The overall level of Knowledge | **Correct** | **351 (86.5)** | **82.8** | **89.4** |
|  | **Incorrect** | **55 (13.5)** | **10.6** | **17.2** |
|  | Total | 406 (100) |  |  |
