# Supplementary material for: Determinants of Male Involvement in the Prevention of Mother‐to‐Child Transmission of HIV in the Bamenda Health District, Cameroon
Source: J Trop Med. 2026 Jul 27;2026:9721872. doi: 10.1155/jotm/9721872 (PMC13402937; doi:10.1155/jotm/9721872)
Supplement: Supplementary file 1 — Supporting Information Figure 1: Knowledge Level of male partners on PMTCT of HIV at the Bamenda Health District from June to September 2020. Supporting Table 1: Knowledge of PMTCT. Supporting Table 2: Attitudes of Men on PMTCT. Supporting Table 3: Practices. [file JOTM-2026-9721872-s001.zip › Supplemental table 2 -Attitude.docx]

**SUPPLEMENTAL TABLE 2 – ATTITUDES OF MEN ON PMTCT**

**Attitude of men on MI in PMTCT in the Bamenda Health District from June to September 2020 (SA - Strongly agree, A - agree, N - neutral, DA- disagree, SDA - strongly disagree)**

| **Variables** | **SA**  **n(%)** | **A**  **n(%)** | **N**  **n(%)** | **DA**  **n(%)** | **SDA**  **n(%)** | **Mean** |
| --- | --- | --- | --- | --- | --- | --- |
| Men should accompany their pregnant partners to ANC/PMTCT | 176 (43.3) | 159 (39.2) | 26 (6.4) | 30 (7.4) | 15 (3.7) | 4.12 |
| Pregnant woman should be tested for HIV even if her partner disagrees | 167 (41.1) | 144 (35.5) | 66 (16.3) | 11 (2.7) | 18(4.4) | 4.06 |
| Discussing HIV testing during pregnancy, delivery and breastfeeding with men is a taboo | 46 (11.3) | 26 (6.4) | 56 (13.8) | 80 (19.7) | 198 (48.8) | 3.88 |
| Couples should be tested together for HIV during ANC follow up even if they are faithful to each other | 197 (48.5) | 128 (31.5) | 37 (9.1) | 32 (7.9) | 12 (3.0) | 4.13 |
| It is enough for less busy family members/relatives to accompany the pregnant woman for ANC clinic | 34 (8.3) | 124 (30.5) | 107 (26.4) | 90 (22.2) | 51 (12.6) | 3.00 |
| An HIV test of a pregnant woman indirectly confirms HIV status of the partner | 21 (5.2) | 76 (18.7) | 56 (13.8) | 122 (30.0) | 131 (32.3) | 3.65 |
| HIV positive pregnant women should be divorced | 18 (4.4) | 26 (6.4) | 70 (17.2) | 109 (26.8) | 183 (45.1) | 4.02 |

| Using condoms reduces the chances of mother-to-child transmission of HIV | 90 (22.2) | 128 (31.5) | 63 (15.5) | 77 (19.0) | 48 (11.8) | 2.67 |
| --- | --- | --- | --- | --- | --- | --- |
| Positive test result of a female partner proves she is unfaithful | 23 (5.7) | 28 (6.9) | 71 (17.5) | 167 (41.1) | 117 (28.8) | 3.81 |
| ANC/PMTCT clinics should provide services for women and children only | 32 (7.8) | 45 (11.1) | 79 (19.5) | 160 (39.4) | 90 (22.2) | 3.51 |
| Women should discuss with male partners what is taught in ANC | 132 (32.5) | 137 (33.7) | 61 (15.0) | 45 (11.1) | 31 (7.6) | 3.71 |
| Postpone HIV testing to post-delivery as pregnancy itself is stressful | 64 (15.8) | 53 (13.1) | 41 (10.1) | 102 (25.1) | 146 (36.0) | 3.53 |

Results showed that most participants had positive attitudes towards male involvement in PMTCT. About four-fifths, 335 (82.5%) of the participants were for the fact that men should accompany their pregnant partners to ANC/PMTCT, while 311 (76.6%) said pregnant women should be tested for HIV even if the partner disagrees. 325 (80%) agreed to couples being counseled and tested together for HIV. Three-fifths 253 (62.3%) had a positive attitude that the HIV result of the pregnant woman does not confirm the HIV status of their partner. Just above half (53.7%) had a positive attitude towards the use of condoms in the reduction of mother-to-child transmission of HIV.
