# Supplementary material for: Determinants of Male Involvement in the Prevention of Mother‐to‐Child Transmission of HIV in the Bamenda Health District, Cameroon
Source: J Trop Med. 2026 Jul 27;2026:9721872. doi: 10.1155/jotm/9721872 (PMC13402937; doi:10.1155/jotm/9721872)
Supplement: Supplementary file 1 — Supporting Information Figure 1: Knowledge Level of male partners on PMTCT of HIV at the Bamenda Health District from June to September 2020. Supporting Table 1: Knowledge of PMTCT. Supporting Table 2: Attitudes of Men on PMTCT. Supporting Table 3: Practices. [file JOTM-2026-9721872-s001.zip › Supplemental table 3 - Practice.docx]

**SUPPLEMENTAL TABLE 3 – PRACTICES**

**Practices of male partner involvement in PMTCT in the Bamenda Health District from June to September 2020**

| **Variables** | **Parameters** | **N (%)** | **Confidence Interval** | |
| --- | --- | --- | --- | --- |
|  |  |  | **Lower** | **Upper** |
| Self-initiated discussion on the importance of PMTCT during the partner’s pregnancy | Yes | 219 (53.9) | 49.0 | 58.6 |
|  | No | 187 (46.1) | 39.9 | 52.2 |
|  | Total | 406 (100) |  |  |
| Request HIV testing for a partner during pregnancy | Yes | 251 (61.8) | 57.1 | 66.6 |
|  | No | 155 (38.2) | 32.0 | 44.5 |
|  | Total | 406 (100) |  |  |
| Ask partner about information from ANC follow-up | Yes | 294 (72.4) | 67.7 | 76.8 |
|  | No | 112 (27.6) | 22.4 | 33.2 |
|  | Total | 406 (100) |  |  |
| Reminded partner of ANC follow-up scheduled | Yes | 317 (78.1) | 73.9 | 82.0 |
|  | No | 89 (21.9) | 16.7 | 28.1 |
|  | Total | 406 (100) |  |  |
| Cover medical expenses (bills) during ANC follow-up during the pregnancy | Yes | 346 (85.2) | 81.5 | 88.7 |
|  | No | 60 (14.8) | 10.4 | 19.7 |
|  | Total | 406 (100) |  |  |
| Accompany partner at least once for ANC clinic | Yes | 263 (64.8) | 60.4 | 69.2 |
|  | No | 143 (35.2) | 30.8 | 39.6 |
|  | Total | 406 (100) |  |  |
| Entered the ANC room when they accompanied their partners | Yes | 140 (53.2) | 47.5 | 59.6 |
|  | No | 123 (46.8) | 40.4 | 52.5 |
|  | Total | 263 (100) |  |  |
| Counseled and tested during spouse’s pregnancy | Yes | 193 (47.5) | 42.9 | 52.5 |
|  | No | 213 (52.5) | 47.0 | 58.2 |
|  | Total | 406 (100) |  |  |
| Counseled and tested together with spouse during pregnancy | Yes | 158 (81.8) | 74.6 | 89.1 |
|  | No | 35 (18.1) | 13.0 | 23.8 |
|  | Total | 193 (100) |  |  |
| Reveal results to each other | Yes | 144 (92.9) | 88.4 | 96.8 |
|  | No | 11 (7.1) | 3.2 | 11.6 |
|  | Total | 155 (100) |  |  |
| Assisted partner in making feeding choices | Yes | 283 (69.7) | 65.3 | 74.1 |
|  | No | 121 (26.8) | 24.4 | 36.2 |
|  | Total | 406 (100) |  |  |
| Use a condom with your partner during pregnancy | Yes | 149 (36.7) | 31.8 | 41.4 |
|  | No | 257 (63.3) | 58.6 | 68.2 |
|  | Total | 406 (100) |  |  |
| Level of Male Involvement | Involved | 173 (42.6) | 47.6 | 57.6 |
|  | Not involved | 233 (57.4) | 42.4 | 52.4 |
|  | Total | 406 (100) |  |  |
